# Whence the beardogs? Reappraisal of the Middle to Late Eocene ‘*Miacis*’ from Texas, USA, and the origin of Amphicyonidae (Mammalia, Carnivora)

**DOI:** 10.1098/rsos.160518

**Published:** 2016-10-12

**Authors:** Susumu Tomiya, Zhijie Jack Tseng

**Affiliations:** 1 Integrative Research and Ganz Family Collections Centers, Field Museum of Natural History, Chicago, IL 60605, USA; 2 University of California Museum of Paleontology, Berkeley, CA 94720, USA; 3 Division of Paleontology, American Museum of Natural History, New York, NY 10024, USA; 4 Department of Pathology and Anatomical Sciences, Jacobs School of Medicine and Biomedical Sciences, University at Buffalo, Buffalo, NY 14214, USA

**Keywords:** Amphicyonidae, Middle Eocene, Carnivora, phylogeny, *Miacis*, Caniformia

## Abstract

The Middle to Late Eocene sediments of Texas have yielded a wealth of fossil material that offers a rare window on a diverse and highly endemic mammalian fauna from that time in the southern part of North America. These faunal data are particularly significant because the narrative of mammalian evolution in the Paleogene of North America has traditionally been dominated by taxa that are known from higher latitudes, primarily in the Rocky Mountain and northern Great Plains regions. Here we report on the affinities of two peculiar carnivoraforms from the Chambers Tuff of Trans-Pecos, Texas, that were first described 30 years ago as *Miacis cognitus* and *M. australis*. Re-examination of previously described specimens and their inclusion in a cladistic analysis revealed the two taxa to be diminutive basal amphicyonids; as such, they are assigned to new genera *Gustafsonia* and *Angelarctocyon*, respectively. These two taxa fill in some of the morphological gaps between the earliest-known amphicyonid genus, *Daphoenus*, and other Middle-Eocene carnivoraforms, and lend additional support for a basal caniform position of the beardogs outside the Canoidea. The amphicyonid lineage had evidently given rise to at least five rather distinct forms by the end of the Middle Eocene. Their precise geographical origin remains uncertain, but it is plausible that southern North America served as an important stage for a very early phase of amphicyonid radiation.

## 1. Introduction

The extinct family Amphicyonidae, commonly called the ‘beardogs’, represents a major group of caniform carnivorans with a rich evolutionary history of over 30 Myr extending from the Middle Eocene to the Late Miocene. During the course of their evolution, amphicyonids spread to all northern continents as well as Africa, and attained formidable ecomorphological diversity that included not only the iconic bear-like forms but also small fox-sized animals and wolf-like pursuit predators [1–6]. While amphicyonid systematics has a long history of study, the origin and initial evolution of the group during the Eocene have long remained nebulous. Examination of the holotype of *Miacis australis* Gustafson, 1986, from the Middle-Eocene portion of the Chambers Tuff of Texas, USA (see [7] for geologic context), prompted the first author to reassess its affinity with other early carnivoraforms, including *Miacis cognitus* Gustafson, 1986, from a higher stratigraphic level in the same formation. The phylogenetic affinities of these two taxa turn out to be eminently relevant to the question of amphicyonid origin as they exhibit combinations of primitive carnivoraform characters—which led to their original assignment to the genus *Miacis* [8]—with some derived amphicyonid conditions. Since 1986, several cladistic analyses of early carnivoraforms have recovered *M. cognitus* as a close relative of the earliest-known amphicyonid, *Daphoenus* (e.g. [9,10]), but the systematic position of the former has not been revised or discussed in detail. Based on a new cladistic analysis that included a larger sample of early amphicyonids than in previous studies, we propose here reassignment of *M. australis* and *M. cognitus* to new genera within the Amphicyonidae. We discuss the results of our analyses and their implications on the origin of beardogs—‘animals of extraordinary structural characteristics and of remarkable interest’ in the words of Scott & Jepsen ([11], p. 76)—with the emphasis on the group's Middle-Eocene fossil record.

### 1.1. Abbreviations

*Institutional abbreviations*—AMNH FM, Fossil Mammal Collection, American Museum of Natural History, New York, NY, USA; CM, Carnegie Museum of Natural History, Pittsburgh, PA, USA; F:AM, Frick Collection, American Museum of Natural History, New York, NY, USA; FMNH, Field Museum of Natural History, Chicago, IL, USA; MNHN, Muséum national d'histoire naturelle, Paris, France; NMB, Naturhistorisches Museum Basel, Basel, Switzerland; OMNH, Sam Noble Oklahoma Museum of Natural History, University of Oklahoma, Norman, OK, USA; SDSNH, San Diego Museum of Natural History, San Diego, CA, USA; SMNH, Saskatchewan Museum of Natural History (presently Royal Saskatchewan Museum), Regina, Canada; TMM, Texas Memorial Museum (in collection of the Vertebrate Paleontology Laboratory, University of Texas at Austin), Austin, TX, USA; UCMP, University of California Museum of Paleontology, Berkeley, CA, USA; UNSM, University of Nebraska State Museum, Lincoln, NE, USA; USNM, United States National Museum of Natural History, Washington, DC, USA; YPM, Yale Peabody Museum, New Haven, CT, USA.

*Other abbreviations*—CI, ensemble consistency index; HRXCT, high-resolution X-ray computed tomography; l.f., local fauna(s); Ma, mega-annum (million years) ago; MPT, most-parsimonious tree; NALMA, North American land mammal ‘age’; RI, ensemble retention index.

## 2. Material and methods

The dental terminology follows Van Valen [12], Flynn & Galiano [13] and Tomiya [14], and the basicranial anatomical terminology follows Wang & Tedford [9]. Lengths and angles were measured on digital photographs using the program tpsDIG2 [15]. Dental measurements reported in Gustafson [8] are not duplicated here. The ages of geomagnetic polarity chrons and subchrons follow the timescale of Gee & Kent [16]. The electronic supplementary materials are available from the Dryad Digital Repository (http://dx.doi.org/10.5061/dryad.5cb57) [17]. New nomenclatural acts are registered in ZooBank, the official registry of Zoological Nomenclature according to the International Commission on Zoological Nomenclature, under the publication LSID: urn:lsid:zoobank.org:pub:E198C2C7-6100-4A71-8248-73AF13CB8BEA.

### 2.1. Cladistic analysis

Phylogenetic definitions of carnivoramorphan higher taxa follow Bryant [18] and Flynn *et al*. [19]. The ‘amphicynodont’ genera *Parictis*, *Subparictis*, *Amphicynodon* and *Pachycynodon* are regarded as basal arctoids with possible affiliations with ursidans (cf. [20–26]), and cephalogalines such as *Cephalogale* are treated as hemicyonine stem ursids [27,28]. To date, no comprehensive phylogenetic-systematic assessment of the Amphicyonidae has been conducted that (i) is founded on computer-based cladistic analysis and (ii) places the group within a broad range of caniform carnivorans (but see Wyss & Flynn [29] for family-level analysis, Hunt [2] for hypothesized genus-level relationships, and Viranta [5] and Peigné [30] for cladograms of post-Eocene amphicyonines). Pending such a study, we provisionally adopt a node-based definition of the Amphicyonidae as a clade consisting of the most-recent common ancestor of *Daphoenus vetus* Leidy [31] and *Cynodictis lacustris* Gervais [32], and all of its descendants (cf. [2,33]).

To test the amphicyonid affinities of *M. australis* and *M. cognitus*, cladistic analyses were conducted with the program TNT v. 1.1 [34,35]. *Oodectes herpestoides* from the Bridgerian North American land mammal ‘age’ (NALMA) was selected as the outgroup taxon because previous cladistic studies consistently placed it near the base of Carnivoraformes (e.g. [10,36]). The most-parsimonious trees were heuristically searched for using the ‘traditional search’ function of the program with the tree-bisection-and-reconnection algorithm and 3000 random-addition sequence replicates (33 trees saved per replicate). Under the program's settings, the node collapsing rule was set to ‘max. length = 0’. Bremer support values and bootstrap support values (with 2000 pseudo-replicates of the character matrix) for internal nodes were obtained using the same program. The ensemble consistency index (CI; [37]) and ensemble retention index (RI; [38]) for the most-parsimonious trees were calculated in the program Mesquite v. 3.04 [39].

The character matrix analysed here (electronic supplementary material, S1) consists of 28 operational taxonomic units (OTUs) and 108 morphological characters, 73 of which are parsimony-informative. A large portion of the character matrix (Characters 1–99) was adopted from those of Wesley-Hunt & Flynn [10] (with modifications described in Tomiya [14] and appendix B) and Tomiya [14]. ‘*Stenogale*’ *julieni* in these previous studies is reported here as *Viretictis julieni* [40]. Given the scope of this study, only those carnivoraforms whose (i) dental and basicranial features are well documented in the literature and (ii) temporal ranges were no younger than the Early Miocene were included in the analysis to minimize homoplasic noise. Unfortunately, few to no postcranial data from securely identified specimens are available for a majority of the early carnivorans included in this study; as such, our analysis relies heavily on craniodental traits. Identification of crown groups is based on the well-established canid affinities of *Hesperocyon gregarius* and *Otarocyon macdonaldi* [41,42], arctoid affinities of *Amphicticeps shackelfordi*, cephalogaline stem ursids, *Mustelavus priscus*, *Broiliana nobilis*, *Plesictis genettoides* and *Zodiolestes daimonelixensis* [24,25,43,44], and feliform affinities of *Palaeoprionodon lamandini*, *V. julieni* and *Proailurus lemanensis* [45]. The states of Character 40 were ordered (cf. [10]), and Character 43 was eliminated as in Spaulding & Flynn [46].

In addition, the character matrix for this study newly incorporated five OTUs and 10 characters based on published accounts and personal observation of available specimens (see appendices A and B for details). These OTUs consist of three early North American daphoenines (*Daphoenictis tedfordi*, *Brachyrhynchocyon dodgei* and *Paradaphoenus minimus*), a composite OTU for the purportedly most primitive European amphicyonid, *Cynodictis* [47], and a composite OTU for cephalogaline stem ursids consisting of the Oligocene taxa ‘*Cephalogale*’ *minor* and *Phoberogale gracile* (cf. [28]). The newly added characters replaced Characters 30, 32, 41, 52, 85 and 86 of Wesley-Hunt & Flynn [10] (appendix B).

### 2.2. Visualization and measurement of basioccipital embayment in *Miacis cognitus*

The basioccipital embayment—a key character in our cladistic analysis—is externally invisible for the holotype TMM 40209-200 of *M. cognitus*. Given this, its extent was reconstructed from micro-computed tomography (micro-CT) scans produced by the University of Texas High-Resolution X-ray CT Facility for the Digital Morphology library (http://digimorph.org). The scans were imported into Mimics Research v. 18 (Materialise, Belgium) as a 16-bit image stack (scan parameters available at http://digimorph.org/specimens/Miacis_cognitus/). A grey-value threshold of 36 264–56 111 was created to highlight the bones of the basicranial region and to digitally eliminate the surrounding sediment matrix. Another threshold of 20 228–56 111 was used to generate an overall cranium region selection. Both threshold regions were reconstructed into three-dimensional models and exported as binary STL files. The model files were imported into Geomagic Studio 10 (3D Systems, South Carolina, USA), and the basioccipital embayment was delineated from the basicranial model and isolated as individual surfaces. The whole-cranium model and the embayment regions were then combined to show the location of the embayment relative to the basicranium and rest of the cranium. Mediolateral widths and dorsoventral heights of the embayment were measured in Geomagic Studio and verified in Mimics Research.

## 3. Results

### 3.1. Cladistic relationships

**Figure 1.**
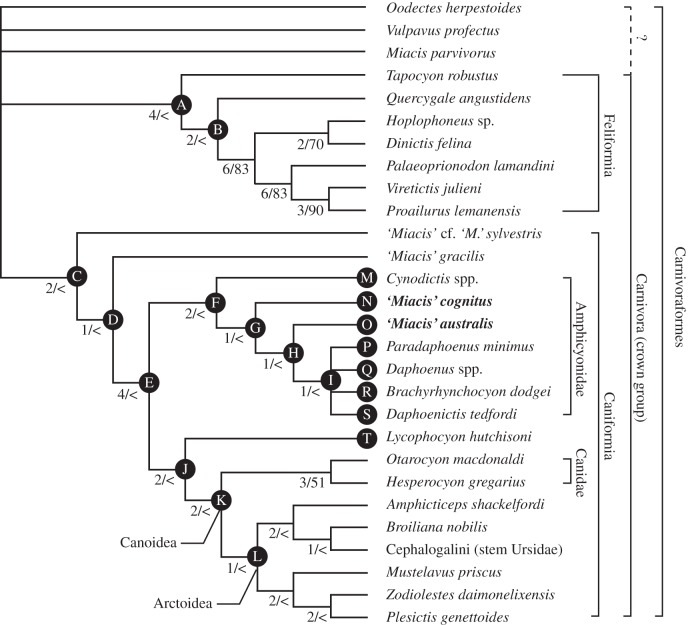
Cladistic positions of ‘*Miacis*’ *australis* and ‘*Miacis*’ *cognitus*. Strict consensus of 27 most-parsimonious trees. Branches are labelled with Bremer support values followed by bootstrap support values (‘<’ for less than 50% support). See text and appendix C for apomorphies associated with labelled nodes.

The cladistic analysis recovered 27 equally most-parsimonious trees (MPTs; tree length = 272 steps, CI = 0.396, RI = 0.624; electronic supplementary material, S1). Apomorphies for key nodes are reported in appendix C. The polytomy at the root of strict-consensus tree (figure 1) results from the absence of unambiguous synapomorphy for the ingroup when *O. herpestoides* is designated as the sole outgroup taxon. Two other North American Bridgerian carnivoraforms have ambiguous relationships with each other and with the rest of the ingroup: *Vulpavus profectus* is found either outside the crown Carnivora (18 of 27 MPTs) or as the earliest-splitting member of Feliformia (9 of 27 MPTs), whereas *Miacis parvivorus* is located outside the crown Carnivora in all MPTs regardless of its uncertain relationship with *V*. *profectus*. As a consequence, the point of origin for the crown Carnivora cannot be precisely determined on the strict-consensus tree. Still, the Feliformia and the Caniformia are recovered as separate clades. In the former (figure 1, Node A), *Tapocyon robustus*, known from the Uintan NALMA of the Rocky Mountain region and southern California, and *Quercygale angustidens*, a Late-Eocene taxon from Europe (note the genus is known as early as the Early Eocene; [48]), are allied with nimravids and the unquestionable feliforms *P. lamandini*, *V. julieni* and *Pr. lemanensis*. On the other hand, the caniform clade (figure 1, Node C) includes the Bridgerian ‘*Miacis*’ cf. ‘*M*.’ *sylvestris* and the Uintan ‘*M*.’ *gracilis*.

The strict-consensus tree supports an amphicyonid clade as a basal caniform group that is positioned outside the crown-group Canoidea. This clade (figure 1, Node F), which includes *Cynodictis* spp., ‘*Miacis*’ *cognitus* and ‘*M*.’ *australis* in addition to the North American Paleogene taxa that have traditionally been recognized as daphoenine amphicyonids, is associated with the following four synapomorphies: (i) reduced paroccipital process (Character 9, State 1; state unknown in four out of seven taxa); (ii) suprameatal fossa absent (Character 24, State 0; state unknown in ‘*M.*’ *australis*); (iii) ‘extremely deep’ (see comments below) basioccipital embayment for the inferior petrosal sinus (Character 31, State 2); (iv) p4 posterior accessory cuspulid descends steeply, giving it a step-like (rather than parabolic or pointed) outline in profile (Character AC7, State 2; state unknown in ‘*M.*’ *cognitus* and shifts to State 1 in *D. tedfordi*; figure 6*c*,*d*). Constrained tree searches show that alternative placement of the amphicyonids within the crown-group Canoidea would increase the tree length by three to five additional steps (table 1). Likewise, six to seven additional steps must be invoked when either ‘*Miacis*’ *australis* or ‘*M*.’ *cognitus* is constrained to form a sister-taxon relationship with the genotypic species of *Miacis*, *M. parvivorus*. Given these results, ‘*M.*’ *cognitus* and ‘*M*.’ *australis* are formally assigned to new genera of basal amphicyonids below.

**Table 1.** Most-parsimonious tree statistics for alternative placements of the Amphicyonidae.

exclusively monophyletic group enforced in constrained tree search	Tree length	CI	RI
none (unconstrained search resulting in figure 1)	272	0.396	0.624
Amphicyonidae^a^ + *Lycophocyon hutchisoni*	274	0.392	0.618
Amphicyonidae^a^ within or sister to crown-group Arctoidea	275	0.391	0.616
Amphicyonidae^a^ + Cephalogalini (stem ursids)	275	0.391	0.616
Amphicyonidae^a^ + Cephalogalini + *Broiliana* + *Amphicticeps*	275	0.388	0.611
Amphicyonidae^a^ + Canidae	277	0.389	0.614

^a^Taxonomic composition of the amphicyonid clade (as recovered in the consensus tree from the unconstrained search; figure 1)—but not its internal topology—was held constant across all constrained searches.

Within the amphicyonid clade, the North American taxa (figure 1, Node G) are united by two synapomorphies: (i) M1 lingual cingulum lingually elongate at the anteroposterior level of the apex of protocone and has a sharp lingual margin (Character AC1, State 1; figure 5*c*,*d*); (ii) M2 moderately large to large relative to P4 (Character AC4, State 0). The consensus cladogram also suggests (figure 1, Node H) that ‘*M*.’ *australis* is more closely related to the daphoenines than ‘*M*.’ *cognitus* is, sharing: (i) an elongate rather than circular infraorbital foramen (Character 3, State 0; state unknown in three out of five taxa); and (ii) position of infraorbital foramen above anterior edge of P4 (Character 4, State 1; state unknown in *D. tedfordi*). *Cynodictis* spp., ‘*M*.’ *cognitus* and ‘*M*.’ *australis* are in turn excluded from the clade of remaining North American amphicyonids by their retention of an anteriorly positioned P4 protocone (Character 82, State 1). The cladistic relationships among the traditionally recognized daphoenines are unresolved (figure 1, Node I).

The position of Early-Miocene arctoid *Broiliana* as a sister taxon to the cephalogaline stem ursids is in conflict with a number of previous cladistic studies that have recovered it as a stem procyonid [24–26,44]. We suspect that additional taxa and characters are needed to accurately place this member of a relatively young (entirely Neogene in distribution) caniform group. For the purpose of the present study, however, this is an ancillary point because removal of *Broiliana* had no effect on the topology of consensus tree.

### 3.2. Systematic palaeontology

Class MAMMALIA *sensu* Rowe [49]

 Order CARNIVORA *sensu* Bryant [18]

 unranked clade CANIFORMIA *sensu* Bryant [18]

 Family AMPHICYONIDAE Trouessart, 1885 [50]

 Genus *GUSTAFSONIA*, gen. nov.

 ZooBank LSID (for nomenclatural act): urn:lsid:zoobank.org:act:2A405FB0-990A-46A5-8106-33979C4120C9

 **Type species**—*Gustafsonia cognita*, comb. nov.

 **Diagnosis**—As for type species.

 **Etymology**—Generic name in honour of P. Eric Gustafson, for his contribution to the study of Eocene-Oligocene mammalian carnivores from the Trans-Pecos region of Texas [8], which laid the foundation for the present paper.

 **Distribution**—As for type species.

 **Remarks**—Determination of the subfamilial affiliation of *Gustafsonia* and, indeed, testing for the reality of currently recognized amphicyonid subfamilies [2] would require broader taxonomic sampling than was attempted here. Essential to such effort would be additional work on the taxonomy of species currently assigned to the genus *Cynodictis*, most of which are based on mandibular dental morphology alone [33] and have not been cladistically analysed.

 *Gustafsonia cognita*, comb. nov.

(figure 2)

**Figure 2.**
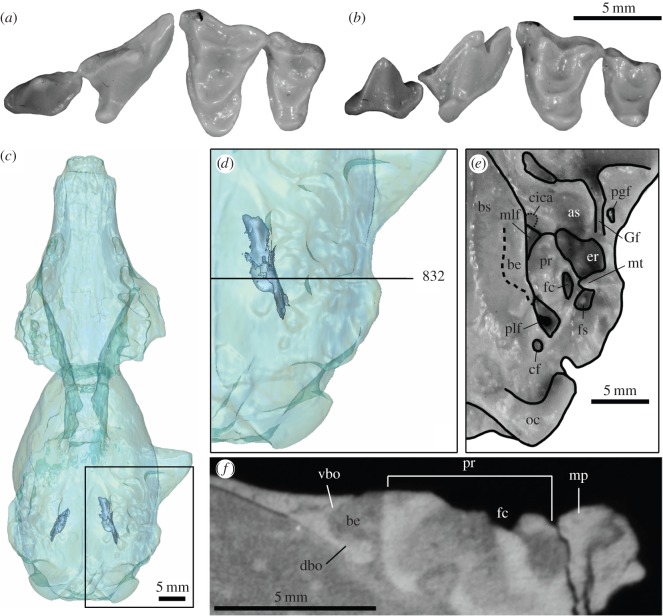
Craniodental morphology of *Gustafsonia cognita*, gen. et comb. nov. (*a*) left P3-M2 of cast of holotype TMM 40209-200 in occlusal view; (*b*) same in oblique lingual view; (*c*) digital three-dimensional reconstruction (see text) of holotype cranium in ventral view, showing locations of basicranial embayment (blue) for inferior petrosal sinuses; (*d*) left basicranial morphology, magnified from boxed area in (*c*) and indicating position of coronal section no. 832 figured in (*f*); (*e*) external ventral view of left basicranial region (cast of holotype; basioccipital embayment is invisible, and its approximate medial extent is marked by dashed line); (*f*) coronal section no. 832 from Digital Morphology Library (see text) showing cross-section through the left auditory region of holotype (ventral to top; lateral to right). Panels (*c*,*d*,*f*) produced with permission from the University of Texas HRXCT Facility. Abbreviations: as, alisphenoid; be, basioccipital embayment for inferior petrosal sinus; bs, basisphenoid; cf, condyloid foramen; cica, canal (dotted line) for anterior loop of internal carotid artery; dbo, dorsal lip of basioccipital; er, epitympanic recess; fc, fenestra cochlea; fs, fossa for stapedius muscle; Gf, Glaserian fissure; mlf, middle lacerate foramen; mp, mastoid process; mt, mastoid tubercle; oc, occipital condyle; pgf, postglenoid fossa; plf, posterior lacerate foramen; pr, petrosal promontorium; vbo, ventral floor of basioccipital.

 *Miacis cognitus* Gustafson, 1986 ([8], p. 38, figs. 23–28) (original description).

 LSID for publication [8]: urn:lsid:zoobank.org:pub:E4457E9C-910C-42CE-9764-70E2C18A5763

 LSID for original nomenclatural act (for ‘*Miacis*’ *cognitus*): urn:lsid:zoobank.org:act:A5C6A848-2273-4E0C-ACAC-9B0F94A24498

 **Holotype**—TMM 40209-200, largely complete cranium (with upper dentition missing C1 and P1 on both sides and left M3); collected by John A. ‘Jack’ Wilson and his field crew from the University of Texas at Austin in 1966.

 **Holotype locality and horizon**—Reeves Bonebed locality (Little Egypt l.f. [7]), Reeves Bonebed, above the upper marker bed [7], Chambers Tuff, Presidio County, Texas [8].

 **Emended Diagnosis (modified from Gustafson ([8], p. 40))**—Differs from: (i) non-amphicyonid carnivoraforms in deep basioccipital embayment for the inferior petrosal sinus (Character 31, State 2; figure 2*c*–*f*), M1 lingual cingulum lingually elongate with a sharp lingual margin (Character AC1, State 1; figure 2*a*,*b*), and where applicable, smaller M3 relative to M2 (M3LxW/M2LxW = 0.16); (ii) other amphicyonids in more gracile dentition and more lingual position of M2 protocone (figure 2*a*,*b*). Further differs from: (i) *M. parvivorus* in the presence of canal on basisphenoid for anterior loop of internal carotid artery (‘cica’ in figure 2*e*; Character 23 State 1), likely medial passage of internal carotid artery suggested by the absence of groove (for promontory artery) on petrosal promontorium (Character 25, State 2; see also ([9], p. 28)), ventral deflection of lateral edge of basioccipital small but present (Character 34, State 1), shorter M1 stylar shelf labial to base of metacone (Character AC2, State 1), the absence of wide trough between mastoid process and paroccipital process (Character 33, State 1; but a narrow, somewhat trough-like depression is present), and greater swelling of lingual M1 cingulum (Character 50, State 2; but note the ‘swelling’ is primarily in the lingual rather than ventral direction); (ii) *Lycophocyon* and canoids in anteriorly elongate and rounded petrosal promontorium (Character 28, State 1); (iii) canoids in smaller lateral flange of basioccipital (Character 34, State 1), M3 present (Character 53, State 0), a steeper angle of M1 postprotocrista (approx. 79° from preprotocrista compared with, e.g. greater than 90° in canids; Character AC3, State 0) and the absence of securely attached ossified auditory bulla (Character AC9, State 0); (iv) *Cynodictis* in more lingual (as opposed to posterolingual) orientation of M1 lingual cingulum, deeper M1 ectoflexus formed by slight labial projection of the posterolabial border of tooth and larger M2 relative to M1 (M2LxW/M1LxW = 0.57 compared with approx. 0.29 for *C. lacustris* ([51], pl. 2, fig. 5)).

 **Distribution**—Known only from the holotype locality; upper portion of the Chambers Tuff, Presidio County, Texas, corresponding to the early Chadronian NALMA [52]. Part of the Little Egypt l.f. [7], which has been assigned to the upper part of Chron 17n [53] (38.11–36.62 Ma) or more precisely to C17n.1n ([52], table 9; 37.47–36.62 Ma). Separately, a maximum age of 38.29 ± 0.16 Ma is given by the oldest of three available ^40^Ar/^39^Ar dates for the Buckshot Ignimbrite, which underlies the Chambers Tuff [52].

 **Remarks**—The specific name becomes *cognita* (Latin feminine participle) when combined with the new genus name, *Gustafsonia*, to match the gender of the latter. *Gustafsonia cognita* is known only from a single cranium. Gustafson [8] figured and described the holotype in detail, and assigned the new species to the genus *Miacis*, emphasizing its generally primitive character (compared with fossil representatives of extant families) and overall similarity to *M. parvivorus.* While its resemblance in dental morphology to the early amphicyonid *Daphoenus* was noted in the original description, an amphicyonid affinity of ‘*M*.’ *cognitus* was rejected on the basis of perceived absence of deep basioccipital embayment. Wang & Tedford [9] reported additional observations on its basicranial anatomy, and noted more derived features of ‘*M*.’ *cognitus* compared with *M*. *parvivorus*, such as more extensive bony coverage of the facial nerve canal and likely medial passage of the internal carotid artery (inferred from the absence of a groove on the petrosal promontorium that would be left by a promontory artery). In a departure from Gustafson's description [8], Wang & Tedford [9] considered the degree of basioccipital embayment in the holotype of ‘*M*.’ *cognitus* indeterminate. Nevertheless, a cladistic analysis by the latter authors suggested a close relationship of ‘*M*.’ *cognitus* to *Daphoenus*. This hypothesis was further supported by subsequent studies that analysed additional taxa and morphological characters (e.g. [10,14,46]).

In its original description, *G. cognita* was described as possessing a ‘narrow’ ([8], p. 42) inferior petrosal sinus, presumably because the approximately 3 mm long contact between the lateral edge of basioccipital ventral floor and the petrosal promontorium leaves little externally visible gap between them. However, high-resolution X-ray computed tomographic (HRXCT) images of TMM 40209-200 [54] reveal substantial embayment of basioccipital above its ventral floor along the line of contact with the promontorium and precisely where it cannot be observed by external inspection (figure 2*c*–*f*). Tracing the embayment from its posterior end forward, it is rather constricted in size immediately anterior to the posterior lacerate foramen but rapidly expands thence to attain a width of 2.0 mm and a height of 1.5 mm at the anteroposterior level of mastoid tubercle; these dimensions are largely maintained anteriorly along the length of the rest of promontorium (figure 2*d*). Thus, at its widest point, the embayment corresponds to as much as approximately 40% of the width of the ventral floor of the basioccipital. This degree of embayment is much greater than seen in the early stem canids *Prohesperocyon wilsoni* and *H. gregarius*, as well as a specimen referred to the possible basal ursidan *Am. shackelfordi* [9,24]. Further, the basicranial structure surrounding the inferior petrosal sinus differs fundamentally from those in more primitive carnivoraforms such as *Miacis parvivorus*, in which the lateral edge of basioccipital floor does not bifurcate into ventral and dorsal lips (contrast [9], fig. 2 with figure 2*f*).

It should be noted that rigorous quantitative scoring of this character (Character 31) as State 1 (deep excavation) or State 2 (‘extremely deep’ excavation as defined by Wesley-Hunt & Flynn [10]) is not possible at present as it would require comparative CT data from additional taxa and allometric consideration. Nevertheless, the result of cladistic analysis reported here is robust to this uncertainty because combining States 1 and 2 into a single state representing deep basioccipital excavation did not alter the topology of consensus tree (electronic supplementary material, S2). The presence of looped internal carotid artery within the inferior petrosal sinus (a feature unique to ursids among extant carnivorans and thought to serve as part of a countercurrent heat exchange system for the blood entering the brain; [55]) cannot be inferred with certainty for *G. cognita* based solely on the deep basioccipital embayment. It is, however, intriguing that the enlargement of inferior petrosal sinus apparently started in the earliest, very small amphicyonids, long before they gave rise to taxa with bear-like proportions.

*Gustafsonia cognita* represents one of the smallest amphicyonids known to date, with a condylobasal length (79.5 mm) approximately 10% shorter than that of the Early-Oligocene *Pa. minimus* from the central Great Plains, which was previously recognized as the smallest North American beardog [3]. A body mass of 2.3 kg (±1 s.e. = [1.4, 3.8]) is estimated from the cranial length using the allometric equation of Van Valkenburgh [56] for mammalian carnivores. Recognition of the genet-sized *G. cognita—*with dentition that lacks obvious signs of dietary specialization—as one of the earliest beardogs accentuates the picture of rapid ecological, if not taxic, diversification of amphicyonids during their earliest-known phase of evolution: the clade gave rise to coyote-sized *Daphoenus* and apparently hypercarnivorous *Daphoenictis* by the Late Eocene (cf. [47]).

 Genus *ANGELARCTOCYON*, gen. nov.

 ZooBank LSID (for nomenclatural act): urn:lsid:zoobank.org:act:A1744550-BAD4-4B2B-8D75-5258BD8E230B

 **Type species**—*Angelarctocyon australis*, comb. nov.

 **Diagnosis**—As for type species.

 **Etymology**—Generic name from Greek ἄγγ*ϵ*λος (angelos = messenger) + ἄρκτος (arctos = bear) + κύων (cyon = dog), in reference to its lingually elongate triangular molars that anticipate those of later amphicyonine amphicyonids.

 **Distribution**—As for type species.

 **Remarks**—As is the case with *Gustafsonia*, additional systematic work is needed to determine the subfamilial affiliation of *Angelarctocyon*.

 *Angelarctocyon australis*, comb. nov.

 (figures 3 and 4)

**Figure 3.**
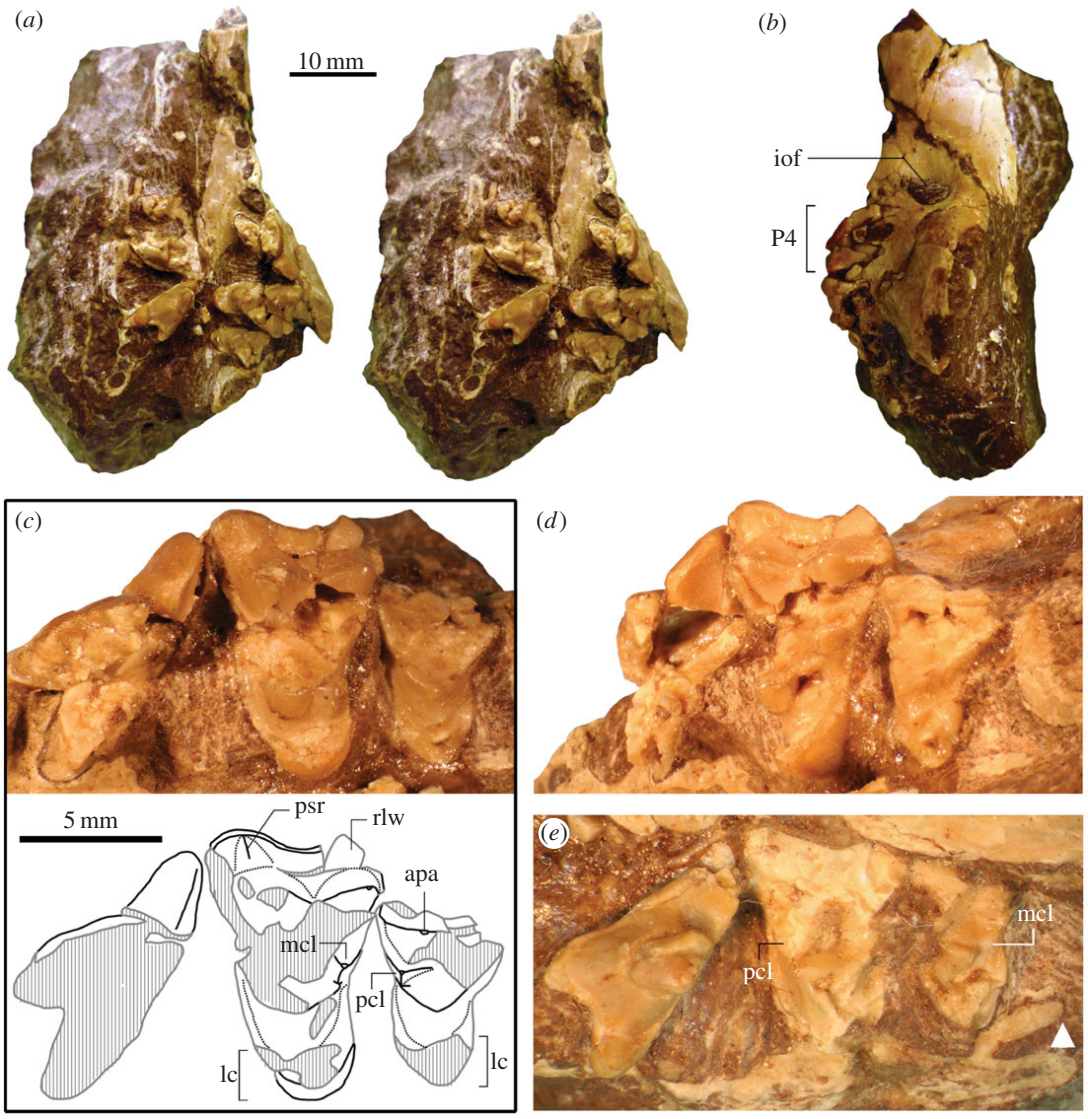
Upper dentition of *Angelarctocyon australis*, gen. et comb. nov. (holotype FMNH PM 423). (*a*) Block of matrix containing right and left maxillae (stereo pair in occlusal view; note anteroposteriorly rotated right maxilla); (*b*) left facial region in lateral view (anterior to top); (*c*) left P4–M2 in occlusal view (shaded areas of line drawing indicating damaged surfaces; black lines represent original borders); (*d*) same in oblique posterior view; (*e*) right P4–M2 (inverted; white triangle points to minimum posterior extent of maxilla). Same scale bar applies to *a* and *b*, and *c*–*e*. Abbreviations: apa, apex of paracone; iof, infraorbital foramen; lc, lingual portion of lingual cingulum; mcl, metaconule; pcl, paraconule; psr, parastylar ridge; rlw, broken piece of rotated labial wall.

**Figure 4.**
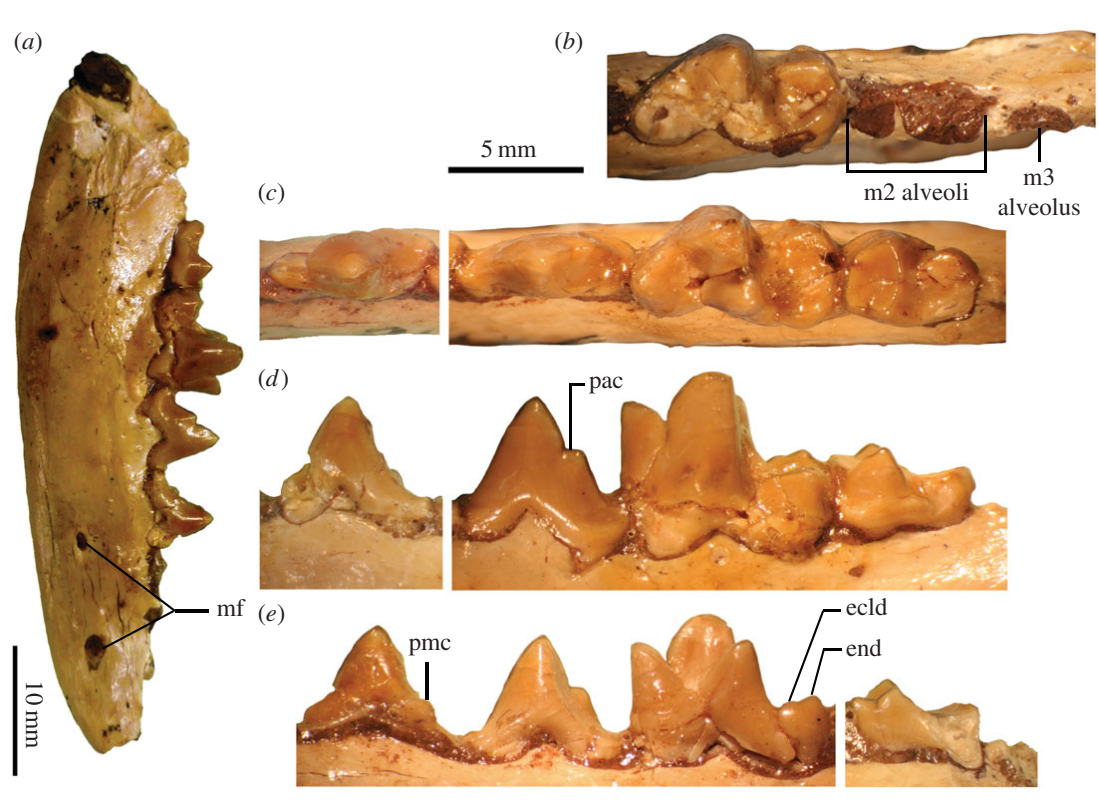
Lower dentition of *Angelarctocyon australis*, gen. et comb. nov. (holotype FMNH PM 423). (*a*) Right dentary in lateral view; (*b*) left m1 and alveoli for m2–3 in occlusal view (inverted); (*c*) right p3–m2 in occlusal view; (*d*) same (inverted) in labial view; (*e*) same in lingual view. Abbreviations: ecld, vestigial entoconulid; end, entoconid; mf, mental foramina; pac, posterior accessory cuspulid; pmc, broken piece of main cuspid originally described as ‘metaconid’ ([8], p. 45). Same scale bar applies to (*b*–*e*).

 *Miacis australis* Gustafson, 1986 ([8], p. 44, fig. 29) (original description).

 LSID for publication [8]: urn:lsid:zoobank.org:pub:E4457E9C-910C-42CE-9764-70E2C18A5763

 LSID for original nomenclatural act (for ‘*Miacis*’ *australis*): urn:lsid:zoobank.org:act:DB2E817E-24A2-4164-A06E-9FE499170196

 **Holotype**—FMNH PM 423, associated right and left maxillae (with nearly complete right P4, heavily damaged right M1–2 and damaged left P4–M2) and right and left dentaries (with right p3–m2 and broken left c1, p3 and m1); collected by Bryan Patterson of FMNH in 1946.

 **Holotype locality and horizon**—Unnamed locality (Porvenir l.f. [7]), horizon 0 to 26.8 m (0 to 88 feet) above the lower marker bed (‘Blue Cliff horizon’ [7]), Chambers Tuff, Presidio County, Texas.

 **Referred specimen**—Unnamed locality ‘near Adobe Springs … 7.6 to 12.8 m (25 to 42 feet) above the Buckshot Ignimbrite, Presidio County, Texas’ ([8], p. 44): TMM 41211-5, dentary fragment with m2.

**Figure 5.**
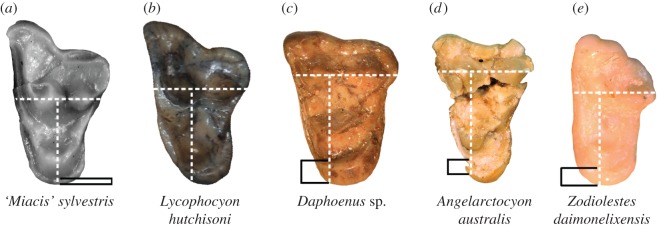
Illustration of character AC1. Form of lingual cingulum (extent demarcated by black lines) was assessed immediately lingual to protocone apex along a labiolingual line (vertical white dashed line) perpendicular to the anteroposterior line running across paracone and metacone (horizontal white dashed line). (*a*) ‘*Miacis*’ cf. ‘*M*.’ *sylvestris* (inverted RM1 of FMNH PM 55953, cast of AMNH FM 129284) showing State 0 (band-like cingulum); (*b*) *Lycophocyon hutchisoni* (inverted RM1 of UCMP 170713) showing State 0 (cingulum nearly confluent with lingual base of protocone and poorly defined, even though it is posterolingually expansive); (*c*) *Daphoenus* sp. (FMNH PM 8694) showing State 1 (inflated and lingually elongate lingual cingulum); (*d*) *Angelarctocyon australis* gen. et comb. nov. (FMNH PM 423) showing State 1 (border between protocone and lingual cingulum is poorly defined, but the latter is clearly elongate in lingual direction); (*e*) *Zodiolestes daimonelixensis* (FMNH P 12032) showing State 2 (moderately inflated cingulum lacking sharp lingual edge unlike in amphicyonids).

 **Emended Diagnosis (modified from Gustafson ([8], p. 44))**—Differs from: (i) non-amphicyonid carnivoraforms in M1 lingual cingulum lingually elongate with a sharp lingual margin (Character AC1, State 1); (ii) other amphicyonids except *G. cognita* in more gracile dentition; (iii) *G. cognita* in more labial (rather than anterior) direction of M1 parastylar region, more robust paracone and metacone of M1, shallow and expansive lingual slope of M1 protocone; and M2 with labiolingually narrower trigon basin (apex of protocone is located approximately halfway between the level of paracone–metacone apices and lingual margin of tooth; figure 3*c*). Further differs from ‘daphoenine’ amphicyonids in greater anteroposterior constriction of lingual portion of M1, more labial direction of M1 parastylar region and larger M2 (length across paracone and metacone is comparable with that on M1). Further differs from: (i) *Miacis parvivorus* in shorter M1 stylar shelf labial to base of metacone (Character AC2, State 1), less pronounced height difference between M1 paracone and metacone, greater swelling of M1 lingual cingulum (Character 50, State 2), more elongate p4 main cuspid across its base (including the portion that bears the posterior accessory cuspulid, this length is approximately 120% of the height of the main cuspid (measured from its apex to the junction of anterior and posterior roots) compared with approximately 73% in USNM 214706), more dorsal position of p4 posterior accessory cuspulid, more open m1 trigonid, more robust m1 entoconid and more elongate m2 (m2 L/W > 1.51 compared with 1.36 for AMNH FM 5019); (ii) basal arctoids (possibly ursidans) *Amphicynodon*, *Pachycynodon* and early stem ursids such as cephalogalines (cf. [21,27,28]) in more acute and anteroposteriorly constricted P4 protocone, relatively taller M1 paracone and metacone, greater anteroposterior constriction of M1 lingual portion, labiolingually wider M1 stylar shelf (not reduced to a mere rim), more triangular outline of M2, more closed m1 trigonid and less reduced m2 paraconid; (iii) early stem ursids in greater lingual elongation and lingual orientation of M1–2 lingual cingula (figure 5*d*).

 **Distribution**—Lower portion of the Chambers Tuff, Presidio County, Texas, corresponding to the late Duchesnean NALMA [52]. Part of the Porvenir l.f. [7], which has been assigned to the lower part of Chron 17n [53] (38.11–36.62 Ma) and ‘most likely’ C17n.3n ([52], p. 235; 38.11–37.92 Ma). Separately, a maximum age of 38.29 ± 0.16 Ma is given by the oldest of three available ^40^Ar/^39^Ar dates for the Buckshot Ignimbrite, which underlies the Chambers Tuff [52].

 **Description**—The following observations on the holotype FMNH PM 423 supplement those of Gustafson [8].

The maxillae are embedded in matrix of hard, tuffaceous sandstone (figure 3*a*). Most of the preserved teeth are extensively damaged. In addition, the dentaries and lower teeth are heavily coated with consolidant, obscuring some of the morphological details. The infraorbital foramen is located directly above the posterior alveolus for P3 (Character 4, State 1; figure 3*b*). Its precise shape is unclear because of the matrix infill, but it appears oblong and taller than wide (Character 3, State 0).

Of the anterior upper dentition, only the root of left canine is preserved. On the left maxilla, diastemata are present between the canine and the single-rooted P1 (approx. 2.2 mm), between P1 and P2 (approx. 3.4 mm) and between P2 and P3 (approx. 1.6 mm), and the spacing of these teeth appears to have been comparable with that in *G. cognita* as well as ‘*Miacis*’ cf. ‘*M*.’ *sylvestris* (FMNH PM 55953 = cast of AMNH FM 129284). The alveoli for P3 suggest posterior widening and triangular outline of the tooth in occlusal view.

The right P4 (inverted in figure 3*e*) is missing its posterolingual base, posterior end of metastylar blade and a small piece of the parastylar area on the anterolabial cingulum; the paracone is broken into pieces and its apical portion is slightly dislodged. The left P4 is heavily damaged except for the labial portion of paracone and the metastylar blade (figure 3*c*,*d*). The parastyle, which is not preserved, is likely to have been absent or very small judging from the surrounding areas. The cingulum is clearly defined along the lingual wall of tooth and anterior to the protocone, but is poorly developed on the labial side of tooth. The preparacrista on the right P4 is a weak ridge that becomes very faint as it approaches the parastylar area. Posterior to the paracone, a deep carnassial notch is followed by a moderately long metastylar blade. The protocone is roughly conical in shape and has a little over a third of the height of paracone.

The right M1 (inverted in figure 3*e*) is extensively damaged except for its anterolingual portion bearing a clearly delineated anterior cingulum (contra absence of such a cingulum reported in the original description ([8], p. 46)) and the lingual portion of trigon basin formed by the labial base of protocone. The left M1 (figure 3*c*,*d*) likewise shows some major breakages, but its basic morphology is discernible. A small piece of the posterolabial wall (‘rlw’ in figure 3*c*) was apparently broken off, rotated lingually and lies on the labial side of metacone; the posterolabial outline of the tooth is thus obscured, but labial projection of that area appears to have been minimal as in *G. cognita* or absent as in *Cynodictis*. The lingual base of paracone and the middle part of the lingual slope of metacone are both missing, but a small portion of the very base of the lingual slope of metacone (immediately labial to the metaconule) is preserved. Also missing are most of the labial slope of protocone, the surface of lingual cingulum immediately lingual to the protocone and the cingulum anterior to the paracone. The preserved portion of protocone is slightly dislodged from the rest of tooth.

A notable feature of the M1 is its prominent lingual cingulum: it extends from the anterior base of the protocone to at least the base of metaconule, from where it seems to merge with the border of tooth (however, it may be partially covered by the matrix). The lingual cingulum (‘lc’ in figure 3*c*) is elongate in lingual direction, and connects smoothly (i.e. without a sharp border) with the lingual to posterolingual base of protocone, forming a concave slope (figure 3*d*). The very apex of protocone is missing, but appears to have been located at about the same anteroposterior level as that of the paracone. The preserved portion of protocone reaches approximately the same height as the paracone. The stylar shelf is very limited in labiolingual width (though not as much as in stem ursids) and is abutted by the steep labial slopes of the paracone and metacone. The low and not particularly sharp parastylar ridge (‘psr’ in figure 3*c*) runs from near the labial base of preparacrista to the labial border of tooth in nearly labiolingual direction. The centrocrista is rather sharp, while the postmetacrista is weakly defined. The paracone (taking into account its missing tip) and the metacone have subequal heights and comparable anteroposterior lengths; both of these cusps are more robust than in *G. cognita*. The paraconule, while completely missing from the left M1, appears to have been well defined, judging from its still fairly prominent remnant on the right M1 (‘pcl’ in figure 3*e*). The metaconule (‘mcl’ in figure 3*c*) is low in height but clearly recognizable, having a somewhat angular appearance. The posterolabial edge of the metaconule gives rise to a narrow cingulum-like band immediately posterior to the metacone. The trigon basin as seen on the right M1 is moderately deep.

The right M2 (figure 3*e*; inverted) is mostly broken except for the trigon basin and the apices of metacone and protocone; the paracone and the lingual cingulum are largely broken off. The left M2 (figure 3*c*,*d*) is missing the labial margin, posterolingual portion of the paracone, and most of the metacone; the lingual portion of the trigon basin is intact, including the paraconule and much of the protocone. The lingual cingulum together with the very shallow lingual slope of the protocone makes the lingual portion of M2 notably elongate. As in the M1, no sharp boundary exists between the lingual base of protocone and the surrounding cingulum, at least in preserved portions; this condition is frequently seen in amphicyonids but not in other caniforms [57]. The protocone is low in height and bears an essentially flat, shallow (and consequently rather long) labial slope that forms a large part of the gently concave trigon basin. Although unclear from the illustration in Gustafson ([8], fig. 29*a*), the apex of paracone is preserved (‘apa’ in figure 3*c*), revealing the paracone to be low and not much taller than the M1 metaconule. The preparacrista is not particularly sharp. The paraconule (‘pcl’ in figure 3*c*) is small with a somewhat angular appearance; it is separated from the protocone by a small notch. An anterior cingulum is present anterior to paracone. The metacone on the right M2 (figure 3*e*) has roughly the same height as the protocone. A very small metaconule (originally described as ‘indistinct’ ([8], p. 45)) is present on the right M2 (‘mcl’ in figure 3*e*); it is recognizable as a slight bulge on the posterior border of the trigon basin lingual to the base of metacone, from which it is separated by a superficial wrinkle.

No M3 is preserved in the holotype, and no M3 alveolus is visible such that its presence was originally considered indeterminate [8]. However, the presence of M3 is almost certain based on the minimum posterior extent of the right maxilla behind (and lingual to) the M2 (white arrow in figure 3*e*).

The dentary (figure 4*a*) is slender (depth below right m1 = 12.0 mm; measured on the lingual side below the paraconid–metaconid junction) with a relatively straight ventral border. The anterior and posterior mental foramina are located below p1 and below the anterior border of p3, respectively. The left c1 measures 4.2 mm in length and 2.8 mm in width at the base of crown; much of the crown above that level is broken off.

The p3 (figure 4*a*,*c*–*e*) is missing its anterolabial portion, and the posterolingual portion is broken into at least two pieces that are held together by adhesive. The broken piece on the lingual side (‘pmc’ in figure 4*e*) appears to be somewhat dislodged, presumably giving it a false appearance of a ‘very small metaconid on the lingual side of the posterior slope of the protoconid’ reported by Gustafson ([8], p. 45). The lingual position of this apparent ‘metaconid’ is, in fact, inconsistent with where an accessory cuspulid is normally located on a carnivoraform p3 (and where it is located on the p4 of FMNH PM 423), which is on a ridge that runs on the labial side of the posterior slope of the main cuspid; thus, there is no plausible sign of a posterior accessory cuspulid or a ‘metaconid’ on the p3 of *A. australis*. The anterior and posterior cingulids do not appear to form any appreciable cuspulids, either, similar to early daphoenines and arctoids but in contrast with other early carnivoraforms such as *Lycophocyon hutchisoni* and *H. gregarius*.

**Figure 6.**
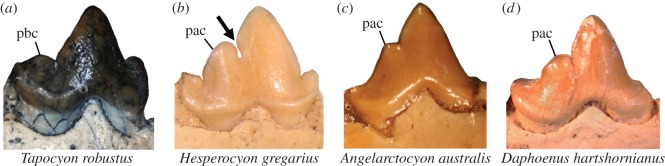
Illustration of Character AC7. (*a*) *Tapocyon robustus* (SDSNH 36000), showing State 0 (trenchant posterior basal cuspulid (pbc) is well developed, but posterior accessory cuspulid (as a structure on the posterior slope of main cuspid) is deemed absent); (*b*) *Hesperocyon gregarius* (FMNH PM 1476), showing State 1 (posterior accessory cuspulid (pac) blade-like with parabolic outline in profile; arrow points to prominent notch); (*c*) *Angelarctocyon australis* gen. et comb. nov. (FMNH PM 423) showing State 2 (small posterior accessory cuspulid with very small notch and steeply descending posterior slope, giving it a step-like appearance in profile); (*d*) *Daphoenus hartshornianus* (FMNH UM 743, inverted) showing State 2 (posterior accessory cuspulid more bulbous than blade-like, with a small notch and monotonically descending, step-like (though somewhat rounded) outline in profile). All in labial view.

The p4 (figure 4) is essentially complete except for chipped pieces of enamel on its lingual base. Mirroring the p3, the p4 lacks anterior and posterior cingular cuspulids, again, similar to early daphoenines and arctoids but in contrast with other early carnivoraforms such as *L. hutchisoni* and *Hesperocyon gregarius* (figure 6). The anterior and posterior cingulids themselves are for the most part only weakly demarcated and do not form a shelf or basin of appreciable size; instead, there is at the anterior end of tooth a short, posterolabially inclined slope whose anterolingual margin forms a very short ridge (figure 4*c*). Unlike in *Hesperocyon*, the central cuspid is positioned more centrally along the length of tooth, has a very weakly sigmoidal anterior border (in contrast with the simply bowing border in *H. gregarius*) and is anteroposteriorly more symmetrical in profile. Further, the notch between the main cuspid and the posterior accessory cuspulid is very shallow, and the posterior accessory cuspulid (‘pac’ in figure 4*d*; ‘hypoconid’ of [8]) is short, in contrast with the elongate, blade-like one in *H. gregarius* (figure 6). The step-like appearance in profile of the posterior cuspulid is characteristic of many amphicyonids (although exceptions are seen in e.g. *D. tedfordi*) and early stem ursids such as *Amphicynodon* and some species of *Parictis*; the p3 and p4 of *A. australis*, however, differ from those of typical ‘amphicynodonts’ in being narrower and lacking thick, clearly defined labial and lingual cingulids. The p4 is narrower and more gracile in appearance than that of *H. gregarius*.

The right m1 (figure 4) is mostly intact but shows numerous cracks and at least two holes on the talonid. The metaconid is well developed and has similar dimensions to the paraconid. The openness of the trigonid in occlusal view is intermediate between those in *L. hutchisoni* and *Daphoenus*, and is much less than that in *Hesperocyon*. The apex of protoconid is either worn down or broken off; the cuspid may have originally been approximately 1–2 mm taller. The presence of anterior and labial cingulids cannot be determined because of breakage. The cristid obliqua and the hypoconid are located more labially than in *Hesperocyon* and most daphoenine amphicyonids; consequently, the labial wall of talonid is nearly vertical. The entoconid (‘end’ in figure 4*e*) is rather inflated as is typical in *Daphoenus* but unlike *Hesperocyon*, in which it forms a low, more compressed ridge). The hypoconid and entoconid are subequal in height and together form a deep talonid basin that is valley shaped in posterior view and is posteriorly open. A very short segment of the rim of talonid basin rises slightly in front of the entoconid; this feature (‘ecld’ in figure 4*e*) might correspond to the better-developed entoconulid seen in other caniforms such as *Daphoenus* (e.g. FMNH PM 8694), *Parictis gilpini* (FMNH PM 22405) and *H. gregarius*.

The right m2 (figure 4) is complete except for the broken posterolingual corner. The paraconid is more distinct than in *Hesperocyon*. The size of m2 relative to m1 is considerably larger than in *H. gregarius* (m2LxW/m1LxW = 0.58 compared with 0.41 for *H. gregarius* calculated from species mean dimensions reported in Wang [41]). The trigonid cuspids are separated by three small notches. As in the m1, the cristid obliqua and the hypoconid are labially positioned, although they are very low in height. The labial portion of talonid is rather horizontal and more so than in *L. hutchisoni*. The entoconid and its surrounding areas are broken off. Separate and weakly defined cingulids are present anterolabial and posterolabial to the protoconid. The posterior slope of metaconid that descends onto the talonid is shallow.

The presence of a single-rooted m3 is evident from its alveolus on the left dentary (figure 4*b*). It seems probable that the tooth was less than half the size of m2.

**Remarks**—The original diagnosis for ‘*Miacis*’ *australis* was confined to comparisons with other species of *Miacis* and *Hesperocyon* ([8], p. 44). Re-examination of the holotype and its inclusion in cladistic analysis for the first time support an amphicyonid affinity of this taxon. We consider the precise phylogenetic relationship between *A. australis* and *G. cognita* to be uncertain: their relative positions within the Amphicyonidae as shown in the strict-consensus tree (figure 1) should be viewed with caution because they are separated on the strict-consensus tree only by subtle differences in the position and the shape of infraorbital foramen—characters that are prone to subjective interpretation as well as taphonomic distortion—and because only the P4–M2 can be directly compared between the two taxa, which are known from a total of three specimens. The preserved upper teeth of the two species are nevertheless quite distinct, and we do not see a compelling reason to assign them to the same genus. The two taxa are known from the same geologic formation in the same general area of Trans-Pecos, Texas, but occur at different stratigraphic levels and have been assigned to separate local faunas that, in turn, are biostratigraphically distinguished as late Duchesnean (the Porvenir l.f. with *A. australis*) and early Chadronian (the Little Egypt l.f. with *G. cognita*) in age.

## 4. Discussion

Originally discussed as ‘relicts of the considerable radiation of miacine miacids’ ([8], p. 46), *A. australis* and *G. cognita* are here formally recognized as basal members of the Amphicyonidae that, in fact, provide important insights into the little-known earliest evolutionary phase of a major carnivoran clade. This reinterpretation follows the progress in understanding of early carnivoraform basicranial anatomy and phylogeny that has been achieved over the last 30 years since the time of their first description (e.g. [3,9,10,24,36,44–46]).

With the exception of *Cynodictis*, lingual rather than posterolingual elongation of the M1 is one of the most diagnostic synapomorphies of early amphicyonids that differentiate them from other early caniforms, including stem canids and stem ursids. This feature typically manifests as some combination of well-developed lingual cingulum (directly lingual to the apex of protocone) and extension of the lingual slope of the protocone, which frequently produces a smooth transition from the protocone to the lingual cingulum [57]. In *Cynodictis*, the orientation of elongate M1 lingual cingulum is decidedly posterolingual as in ‘*Miacis*’ *gracilis* (although the latter has a considerably shorter cingulum), canids and several early arctoids including stem ursids; this condition may, therefore, be ancestral to amphicyonids given their phylogenetic position and internal topology. An isolated caniform upper molar (SMNH P661.1492) from the Chadronian Calf Creek l.f. of the Cypress Hills Formation essentially shows this condition but was questionably reported as an M1 of the ‘amphicynodont’ *Subparictis parvus* ([58], fig. 3.3; ‘*Parictis’ parvus* in that publication) and later described as being ‘much more primitive than those of other species of *Subparictis*’ ([22], p. 490). In fact, no unquestionable upper tooth of *Su. parvus* is known, and SMNH P661.1492 more closely resembles the M2 s of *A. australis* (FMNH PM 423) and *Daphoenus* cf. *D. lambei* (OMNH 2953) than the M1 s of other species of *Subparictis*, in which the extension of M1 lingual cingulum is less pronounced and is more posteriorly directed (cf. [20]).

The removal of *G. cognita* from the genus *Miacis* leaves the latter with no specifically determinate or generically secure occurrence in the Late Eocene. Here, it is worth noting that *Miacis* represents perhaps one of the most confused genera in the classification of early carnivoraforms, with at least 19 species having been assigned to the genus (cf. [59–61]). None of them other than the type species *M. parvivorus* from the Bridgerian of North America has received cladistic support for its generic assignment [10,36,46]; as such, the genus as currently recognized defies apomorphic diagnosis. We suspect that the historical tendency of taxonomic workers to place primitive carnivoraforms in *Miacis* has obscured more than it clarified the patterns of their diversification, and await a comprehensive revision of the genus.

### 4.1. Phylogenetic position of the Amphicyonidae

In assessing the affinity of what is still the earliest-known amphicyonid (then considered a canid) genus, *Daphoenus*, Scott and Jepsen remarked in their 1936 paper: ‘This very difficult problem is full of paradoxes and seeming contradictions’ ([11], p. 74). At the heart of the problem was the amalgam of plesiomorphic and homoplasic traits seen in the White River carnivorans, which the authors were well aware of but lacked an analytical framework to dissect. Homoplasy in skeletal morphology is well known among carnivorans (cf. [62–64]), and can become increasingly problematic in phylogenetic inference as the taxic and spatio-temporal scope of analysis expands. Comparison of early members of major clades is thus expected to be more informative for phylogenetic reconstruction than that of more derived members, which tend to be better represented in the fossil record but are separated by longer branches; the latter approach (e.g. [65,66]) may have contributed to the long history of systematic instability surrounding the beardogs, much of it predating widespread use of the cladistic method [2,67–71] (see [5,69] for historical reviews).

To date, computer-based cladistic analyses of early carnivoran phylogeny have included only one to three OTUs representing amphicyonids (e.g. [10,14,29,46]). The results of previous studies variously suggested amphicyonids to be the sister group of the Ursidae [29] or the Canidae [46], or a basal clade outside the Canoidea [10,14]. Though still limited in taxic coverage, this study expands the previous efforts by substantially increasing the sampling of amphicyonids from the earliest phase of their radiation, which is key to deciphering their relationship to other carnivoran clades, and by incorporating hitherto-neglected morphological characters to help resolve their phylogenetic positions.

The identification of amphicyonids as an early diverging group of caniforms outside the Canoidea is in agreement with, and provides additional support for, previous findings by Wesley-Hunt & Flynn [10] and Tomiya [14], and was already considered a possibility 20 years ago [47]. Statistical support values for most of the individual nodes in the recovered consensus tree are low (figure 1), implying both a proportionately small number of traits that represent synapomorphies and already a high degree of homoplasy. Nevertheless, alternative placement of amphicyonids as the sister group to canids, arctoids, or any subgroup of arctoids is clearly less acceptable based on the parsimony criterion (table 1).

The hypothesis of amphicyonid–ursid relationship based on the shared presences of deep basioccipital embayment and postscapular fossa [2,29,72,73] merits additional discussion. The main difficulty with this hypothesis, which is not supported by the present parsimony analysis, is that the earliest-known arctoids including some stem ursids are considerably more derived than the earliest amphicyonids. For example, *Amphicynodon*, *Cephalogale* (a constituent of the cephalogaline OTU in figure 1) and their close relatives from the Oligocene of Eurasia almost always lack the M3 and, where known, possess a more flat petrosal promontorium as well as horizontally expanded auditory (primarily ectotympanic) bullae that differ from the simple crescentic ones in the earliest amphicyonids (cf. [21,24,27,28]). Similarly non-crescentic and extensively ossified bullae are suspected—albeit from poorly preserved material—for an even older amphicynodont, *Campylocynodon personi* from the Late Eocene (Chadronian) of North America [20]. The appearance of sister-taxon relationship may be reinforced when comparing more derived members of the two families, but many of their most conspicuous evolutionary trends (e.g. development of the flask-shaped Type A auditory bulla of Hunt [55] as opposed to its precursors seen in e.g. *Daphoenus* (cf. [73], p. 830, [47], p. 502), enlargement of posterior molars and pronounced body size increase) are known to represent parallelism or convergence [2,47,68,70,73].

An exclusive amphicyonid–ursid relationship, therefore, cannot be supported unless (i) more primitive stem ursids are discovered that reveal additional synapomorphies with the earliest amphicyonids or (ii) the two potential synapomorphies (basioccipital embayment and postscapular fossa) are more heavily weighted in cladistic analysis. However, for carnivoramorphans, the traditional expectation that basicranial anatomy is less prone to homoplasy through ecological adaptations than other craniodental traits (e.g. [69]) is not supported by analysis of empirical data [10]. Indeed, while the degree of basioccipital embayment that characterizes amphicyonids and ursids is not seen in other carnivorans, it should be noted that enlargement of the inferior petrosal sinus also occurred—presumably independently—in mustelidans [10,43]. A quantitative survey of the basioccipital embayment in early carnivorans and greater knowledge of their postcranial and brain morphology (cf. [46,74]) would enable additional testing of the various phylogenetic hypotheses.

### 4.2. Time and place of amphicyonid origin

Before their geographical range extension into Africa and Panama in the Early Miocene, amphicyonids were confined to the Holarctic (cf. [1,75,76]). Their first occurrences in different regions of the Holarctic are reviewed here.

*Simamphicyon helveticus*, known from the late-Middle Eocene of Europe (first occurrence in the mammal reference level MP 16, *ca* 40–37 Ma; [71]; see [77,78] for timescale) has generally been treated as an amphicyonid since the time of its original description ([79]; see also [80] and [67], p. 81), and was regarded as the oldest amphicyonid by Springhorn [71]. However, several authors have expressed doubts as to its amphicyonid affinity [71,81–84]. Indeed, published figures of fragmentary specimens attributed to *S. helveticus* ([81], fig. 5–8; [82], fig. 1–17; [83], fig. 10; [84], text-fig. 17–21) do not exhibit any of the amphicyonid synapomorphies identified in the present study; on the contrary, it retains a labiolingually wide M1 stylar shelf and lacks a lingually elongate M1 lingual cingulum (these features were, in fact, noted by Pictet & Humbert ([80], p. 135) and by Schlosser ([67], p. 81)) unlike all unquestionable amphicyonids.

We concur with Viret's ([81], p. 97) opinion (based on reduced lower premolars and the trenchant m1 talonid) that *S. helveticus* is more closely related to the basal carnivoraform *Uintacyon*. In addition, *S. helveticus* shares notable similarities with the North American Uintan taxon *Miocyon vallisrubrae* (S.T., 2016 personal observation, on a cast of TMM 40165-4, figured as ‘*Uintacyon*’ *scotti* in Gustafson [8] and later reclassified [85]) in the reduced heights of upper-molar cusps, the relatively large M2 that lacks rapid lingual constriction, and the posterolabial orientation of the m1 talonid basin ([84], text-fig. 20). While Viret ([81], p. 97) specifically discounted a close relationship between *S. helveticus* and *Miocyon* (then ‘*Prodaphaenus*’) *scotti* because of the pronounced transverse widening of P4 in the latter species, this feature is lacking in *M. vallisrubrae*, so a close genus-level relationship cannot be rejected on that ground. Consequently, the earliest and the only Eocene occurrences of amphicyonids in Europe are those of *C. lacustris* (first occurrence in MP18, *ca* 36–35 Ma; [33,86]) and possibly *Pseudocyonopsis antiquus* from the Late Eocene, although the latter is more securely known from the Early Oligocene [69,71].

From Asia, Middle-Eocene occurrences of *Cynodictis* in the Lushi Basin ([87], p. 167) and ‘cf. *Cynodictis*’ in the Ulan Shireh Formation ([88], p. 116) of China have been reported in faunal lists. They might represent the earliest records of the Amphicyonidae (see [89] for the ages of assemblages), but we have not been able to verify either identification in the absence of unique identifiers for the pertinent specimens. A revision of the genus *Cynodictis* [33], which restricted its constituents to European species, and frequent misassignment of non-amphicyonid carnivorans to *Cynodictis* (rectified by e.g. [45,57,90]) cast further doubt on the generic identification as well as the amphicyonid affinity of the purported Middle-Eocene ‘*Cynodictis*’. The only other possible occurrences of beardogs in the Middle Eocene of Asia consist of fragmentary teeth (a canine and a possible m1 talonid) from the late-Middle Eocene of Myanmar, and are questionably referred to the family [91]. The earliest definitive amphicyonid occurrences in Asia, then, are from the Late Eocene of China and Mongolia [57,92]. Despite the limited material available for comparison (*Guangxicyon sinoamericanus* is represented by a dentary and two limb elements [92], and the unnamed taxon from Mongolia by an incomplete M2 [57]), these taxa appear to be considerably more derived than the older *Angelarctocyon* and *Gustafsonia* from North America: for example, *Gu. sinoamericanus* is approximately 270% the size of *A. australis* (based on m1 length) and possesses low-crowned lower cheek teeth unlike those of *A. australis* but similar to some European amphicyonine amphicyonids (cf. [92]).

Thus, at present, the only verifiable Middle-Eocene occurrences of the family are from the Duchesnean NALMA (roughly comparable with the Bartonian age) of North America. Chronologically, they can be summarized as follows (see [47,52,93] on the ages of localities): (i) early Duchesnean—*Daphoenus demilo* from Wyoming [94]; (ii) ?middle Duchesnean—*D. demilo* from Saskatchewan [95]; (iii) late Duchesnean—*Daphoenus* cf. *D. lambei*, *A. australis* and cf. *Daphoenictis* sp. from Texas [8,47,96]; (iv) unknown portions of Duchesnean—*D. lambei* from Wyoming & Saskatchewan [47,97]. Other authors, who have proposed a latest-Duchesnean age for the Little Egypt l.f. from the Chambers Tuff [98], would add to this list *G. cognita* and *B. dodgei* (cf. [8]). Curiously, amphicyonids are absent from the latest-Uintan to early-Duchesnean assemblages west of the Rocky Mountain region (e.g. Hancock Quarry l.f. (Clarno Formation) of Oregon, Laguna Riviera and Pearson Ranch l.f. (Santiago and Sespe Formations) of southern California), which include some of the oldest North American occurrences of immigrant carnivore lineages from Eurasia (represented by hyaenodontids *Hemipsalodon* and *Hyaenodon* and a nimravid [99–101]), and of an early non-canoid carnivoraform, *Lycophocyon* [14,102].

The topology of consensus tree, the morphological diversity of Duchesnean taxa and the temporal distribution of fossil occurrences together make it clear that the beardogs had not only originated but also given rise to at least five distinct genera or their precursors (*Cynodictis*, *Gustafsonia*, *Angelarctocyon*, *Daphoenictis* and *Daphoenus*) by the end of Middle Eocene, approximately 37 Ma. The geographical origin of the family, on the other hand, is difficult to infer from the available data. Arguments for and against three alternative scenarios can be summarized as follows:

(1) Europe. The European species *C. lacustris* is more plesiomorphic than the earliest North American amphicyonids in some respects (e.g. greater labial extent of M1 parastylar region, small M2/m2, retention of vestigial anterior cingular cuspulids on lower premolars). However, the complete absence of beardogs from the fairly substantial Middle-Eocene faunal records for Europe (cf. [103,104]) makes a European origin less likely. Detailed investigation of the phylogenetic relationships among various species of *Cynodictis* and Eurasian amphicyonines, which has not been attempted since the work of Teilhard de Chardin [51], may help clarify the geographical context of early amphicyonid evolution.
(2) Asia. Few carnivoraforms were reported—and even fewer were confidently identified—from the Middle Eocene of Asia in the faunal compendium of Russell & Zhai [88], and the poor state of knowledge has not fundamentally changed since then despite some important findings [105]. As a result, there is little empirical evidence for an Asian origin of amphicyonids. Still, recent discoveries of Late-Eocene taxa in China and Mongolia that appear to be more derived toward later amphicyonines than the roughly contemporaneous *Cynodictis* in Europe suggest eastern Eurasia as a potentially important stage for the early diversification of amphicyonids.
(3) North America. The following observations can be listed in support of a North American, and possibly southern North American, origin: (i) all secure Middle-Eocene occurrences of the family are confined to that continent, and four to six species had already appeared in North America before the first occurrence of beardogs anywhere in Eurasia; (ii) two of the earliest-diverging forms (*Gustafsonia* and *Angelarctocyon*) are known from there and are, in fact, known only from deep inside the continent as opposed to at higher latitudes closer to the land connection with Eurasia. A better fossil record compared with Asia and perhaps also Europe may explain the first observation, but the second point is puzzling under the hypothesis of Eurasian origin. However, the cladistic position of European *Cynodictis* in the consensus tree makes a North American origin less than certain.

Noteworthy from a broader biogeographic perspective are the endemic occurrences of some of the earliest (evolutionarily, if not always chronologically) members of emerging mammalian groups in the late Duchesnean to early Chadronian of Trans-Pecos, Texas. They include the most primitive stem canid *Prohesperocyon wilsoni* [41], the ‘anchitheriine’ equid *Mesohippus texanus* [106,107] and the advanced stem primate or basal crown primate *Rooneyia viejaensis* [108,109] in addition to the early amphicyonids listed above. Together with a recent discovery of the first Chadronian fauna in southern Mexico that contained an early jimomyid rodent and a derived chalicothere along with two undetermined caniform species (from published data, we think they might represent amphicyonids given their relatively large sizes) [110], these biogeographic patterns highlight the low-latitude regions within North America as a potential centre of mammalian diversification during the Middle to Late Eocene.

## Figures and Tables

**Figure 7.**
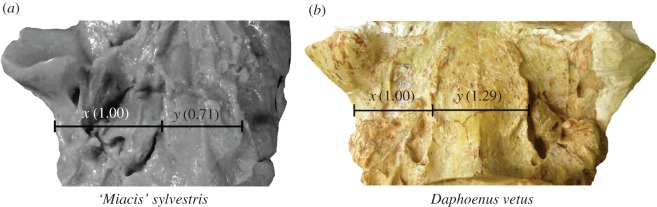
Illustration of Character AC10. Width (*x*) of basicranium lateral to lateral edge of basioccipital ventral floor compared with width (*y*) of basioccipital ventral floor at the level of external auditory meatus. (*a*) State 0 (*y* = 0.71*x* < *x*) in ‘*Miacis*’ cf. ‘*M*.’ *sylvestris* (FMNH PM 55953, cast of AMNH FM 129284); (*b*) State 1 (*y* = 1.29*x* > *x*) in *Daphoenus vetus* (FMNH PM 8388).

## Data accessibility

Character matrix data are available as the electronic supplementary material from the Dryad Digital Repository (http://dx.doi.org/10.5061/dryad.5cb57) [17]. The raw HRXCT images are properties of the University of Texas HRXCT Facility and were used with permission; essentially the same data are publicly accessible at: http://digimorph.org/specimens/Miacis_cognitus/.

## Appendix A. Data sources for cladistic analysis

Specimens listed here are in addition to those listed in Tomiya ([14], app. S1).

**Table:**

OTU	specimen(s) examined	literature source(s)
*Oodectes herpestoides*	AMNH FM 105003 (cast of holotype YPM 11861), AMNH FM 11499	
*Vulpavus profectus*	AMNH FM 12626 (holotype)	
*Miacis parvivorus*	AMNH FM 11500, USNM 214706	
*‘Miacis*’ *gracilis*	AMNH FM 143785 (cast of holotype CM 11900), AMNH FM 104960 (cast of CM 12063)	
*Tapocyon robustus*	FMNH PM 538 (*T*. cf. *T. robustus*), FMNH PM 22735	
*Quercygale angustidens*	UCMP 63172, UCMP 79947 (cast of MNHN F.QU8755)	[111,112]
*Dinictis felina*	FMNH P 12004	[113] (nimravid general)
*Hoplophoneus primaevus*	FMNH UM 420, FMNH UM 474	[113] (nimravid general)
*Palaeoprionodon lamandini*	UCMP 63097	[45,51,67,114]
*Viretictis julieni*		[45,115]
*Proailurus lemanensis*	UCMP 118323, AMNH FM 101931 (cast of holotype MNHN SG3509)	[45,115]
*‘Miacis*’ *australis*	FMNH PM 423 (holotype)	
*‘Miacis*’ *cognitus*	TMM 40209-200 (cast of holotype)	
*Cynodicti*s spp.^a^	*C.* sp.^a^: AMNH FM 10060, UCMP 63170, UCMP 63171, UCMP 63173, UCMP 63175	[3,116] (NMB QV 412)
	*C. lacustris*: AMNH FM 10056, UCMP 62709, UCMP 63054, YPM PU 10614	
*Paradaphoenus minimus*	AMNH FM 39099 (holotype), F:AM 72513, AMNH FM 80223	
*Daphoenictis tedfordi*	F:AM 76205	[117] (F:AM 76205); [118] (USNM 214642); [119] (UNSM 27015)
*Daphoenus* spp.	*D. hartshornianus*: FMNH UM 488, FMNH UM 741, FMNH PM 8694	
	*D. lambei*: OMNH 2953 (cast)	
	*D. vetus*: FMNH UC 477, FMNH UC 1426, FMNH UC 1456, FMNH UM 744, FMNH UM 745, FMNH P 12021	
	*D*. sp.: FMNH PM 20057, FMNH P 26243	
*Brachyrhynchocyon dodgei*	FMNH UC 1755, FMNH PM 20769, FMNH P 12140, UCMP 65673 (cast of CM 9256), USNM 362761	
*Amphicticeps shackelfordi*		[24] (MAE BU.91.9187–90)
Cephalogalini^b^	*Cephalogale* sp.: UCMP 100009 (cast of Musée de Gèneve, Mou.14, figured in ([27], fig. 20 as C. ‘sp. II’))	‘*Cephalogale*’ *minor*: [27]
	*Ph. gracile*: AMNH FM 39298 (cast of Musée de Lyon, St.-G. 795 bis)	*Ph. gracile*: [27] (as ‘*Cephalogale*’ *gracile*)
*Mustelavus priscus*	FMNH UM 722, FMNH PM 46451 (*M*. cf. *M. priscus*)	
*Zodiolestes daimonelixensis*	FMNH P 12032 (holotype)	
*Hesperocyon gregarius*	FMNH UC 496, FMNH PM 1467, FMNH PM 1476, FMNH PM 1478, FMNH PM 1479, FMNH PM 1487, FMNH PM 1488, FMNH PM 1490, FMNH PM 1492, FMNH PM 1493	
*Otarocyon macdonaldi*	AMNH FM 38986 (holotype)	[42]

^a^The composite OTU *Cynodictis* spp. consisted of the Late-Eocene (Priabonian) *C. lacustris* and likely one additional undetermined species of that genus.

^b^See [28] for systematics.

## Appendix B. Additional notes on character matrix data

**Changes to data from previous studies [10,14]**

*Character 25*: The definition of State 2 was slightly modified such that it included all conditions in which there was no evidence for transpromontorial passage of internal carotid artery, without implying a specific alternative position (e.g. inside or outside the auditory bulla) for that artery. Accordingly, the character state for *Daphoenus* spp. (originally scored as unknown [10]) was scored as State 2. While Hunt ([117], p. 1032) listed probable transpromontorial course of internal carotid artery as one of the diagnostic features of the subfamily Daphoeninae, a groove on the promontorium that would indicate such a condition has only been documented for F:AM 76205 of *D. tedfordi* [117].

*Character 30*: This character was excluded from the analysis because of the difficulty in (i) distinguishing between ‘smooth’ (State 0) and ‘roughened’ (State 1) areas on the anteromedial portion of promontorium or tympanic wing of basisphenoid and (ii) interpreting the derived state as evidence for the presence of rostral entotympanic. The problem is reflected in the apparently conflicting states that have been reported for the same taxa in different studies: e.g. viverravid *Protictis schaffi* either has a rugose area on the promontorium ([120], p. 112; interpreted by Wang & Tedford ([9], p. 5) as a probable site of attachment for the rostral entotympanic) or does not ([10]; Character 30 scored as State 0), and the basal carnivoraform *Vu. profectus* either has ([10]; Character 30 scored as State 1) or lacks ([9], p. 5) such an area. The character state for *Daphoenus* was originally scored as State 1 [10], but neither the rostral entotympanic itself nor its attachment site on the promontorium or basisphenoid has been clearly identified in amphicyonids [2]. While the rostral entotympanic was present in all of the extant carnivorans examined by Hunt [55], further anatomical investigation is warranted to determine—if at all possible—its presence among basal carnivoramorphans.

*Character 31*: ‘*Miacis*’ *cognitus* (originally scored as State 0 [10]) was scored as State 2 (extremely deep basioccipital embayment for inferior petrosal sinus) based on HRXCT images of the holotype TMM 40209-200 (see Remarks for *G. cognita* in the Systematic Palaeontology section).

*Character 37*: ‘*Miacis*’ *cognitus* (originally scored as State 1 [10]) was scored as State 0 (fossa for stapedius muscle anteriorly bound by mastoid tubercle) because the observed condition in which the mastoid tubercle is in solid contact with the promontorium (cf. [8], fig. 24) was deemed comparable with that in AMNH FM 129284 of ‘*Miacis*’ cf. ‘*M*.’ *sylvestris* (cf. [9], fig. 4).

*Character 59*: ‘*Miacis*’ *gracilis* (originally scored as unknown [10]) was scored as State 1 (m2 with short talonid and oval outline) because the holotype CM 11900 and a referred specimen CM 12063 clearly show this condition.

*Character 88*: *Mustelavus priscus* (originally scored as State 0 [14]) was scored as State 1 (m3 absent) as all but one of the specimens examined showed the latter condition, and the state in holotype YPM PU 13775 (previously interpreted as having m3 alveolus) was deemed ambiguous.

Characters 60, 61, 63, 66, 67, 78–84, 87 and 88 for *Ot. macdonaldi* (all originally scored as ‘?’ [10]) were newly scored based on the holotype AMNH FM 38986.

**Newly added characters**

AC1 (replaces Character 41). Size and form of M1 lingual cingulum directly lingual to apex of protocone

0: lingual cingulum directly lingual to the apex of protocone is a thin band (as in ‘*Miacis*’ *sylvestris* and some individuals of *Hesperocyon*), or there is no distinct cingulum at that level (common in *Hesperocyon*; also observed in feliforms and ‘cat-like’ forms with vestigial to no lingual cingulum; figure 5*a*,*b*)
1: elongate with relatively sharp lingual margin (common in amphicyonids; figure 5*c*,*d*)
2: moderately developed to elongate with rounded lingual margin (e.g. *Zodiolestes*; figure 5*e*).

AC2. Width of M1 stylar shelf labial to base of metacone

0: wide—subequal to the length of metacone (e.g. ‘*Miacis*’ *sylvestris*)
1: considerably narrower (approx. 1/2 or less) than the length of metacone (e.g. *Hesperocyon*, most amphicyonids)
2: metacone vestigial, base of cusp not clearly defined (e.g. feliforms).

AC3. Orientation of M1 postprotocrista in occlusal view

0: narrow angle (less than 90°) between preprotocrista and postprotocrista, the latter having comparatively labial orientation (e.g. *Miacis parvivorus*, *Gustafsonia*, *Angelarctocyon*)
1: wide angle (greater than or equal to 90°) between preprotocrista and postprotocrista, the latter having comparatively posterior orientation (e.g. *Hesperocyon*, *Daphoenus* (including *D. lambei*, though more pronounced in later species), *Paradaphoenus*)
2: protocone well developed but postprotocrista absent or poorly defined (e.g. *Daphoenictis* and mustelidans)
3: protocone and postprotocrista vestigial (e.g. most feliforms)
  Note Character 85 (presence/absence of m1 talonid) was excluded from analysis as it clearly overlapped with Character AC3.

AC4 (replaces Characters 52 and 86). Size of M2

0: M2 length (at level of lingual base of paracone)/P4 L ≥ 0.40 (moderate–large as is typical in amphicyonids, ursidans, *Broiliana* and some basal carnivoraforms)
1: M2 length (at level of lingual base of paracone)/P4 L = [0.25, 0.40) (small–moderate as in some basal carnivoraforms and *Hesperocyon*)
2: M2 length (at level of lingual base of paracone)/P4 L < 0.25 (very small or absent as in *Tapocyon* and feliforms).

AC5. Size of p3 posterior cingular cuspulid

0: present (may be small as in e.g. *Brachyrhynchocyon*)
1: vestigial to absent (e.g. *Daphoenus*)
2: relatively well developed, tall and blade-like (convex border in profile; e.g. *Hesperocyon*).

AC6. Size of cuspulid/cingular ridge on p4 anterior cingulid

0: small to vestigial but pointed cuspulid present that projects against the descending anterior slope of main cuspid in profile (e.g. ‘*Miacis*’ *sylvestris*, *Lycophocyon*)
1: prominent (e.g. *Tapocyon*, *Hesperocyon*)
2: completely absent (e.g. *A. australis*, *Daphoenus*, mustelidans, *Quercygale*).
  Note *Daphoenus* spp. was scored as State 2, but one of the seven specimens (FMNH UM 741, tentatively identified as *D*. cf. *D. hartshornianus*) examined for this character showed a small cuspulid (State 0); the latter is here considered to be an aberrant condition, but polymorphic scoring may be warranted if such a condition is found to be more prevalent.

AC7. Size and form of p4 posterior accessory cuspulid on posterior slope of main cuspid

0: absent (there may be a posterior basal cuspulid; e.g. feliforms and ‘cat-like’ forms; figure 6*a*)
1: apical portion prominent, somewhat conical to blade-like; posterior slope not very steep (e.g. *Hesperocyon*; figure 6*b*)
2: step-like in profile, with small apical portion and steep posterior slope (e.g. *A. australis*, most individuals of *Daphoenus*; figure 6*c*,*d*).

AC8. Position of m1 hypoconid

0: positioned within the labial 1/4 (greater than 0.75 from lingual side) of the labiolingual width of talonid (measured at the anteroposterior level of the apex of hypoconid; e.g. *Angelarctocyon*, *C. lacustris*)
1: positioned within the lingual 3/4 (less than or equal to 0.75 from lingual side) of the labiolingual width of talonid (e.g. *Daphoenus*, *Daphoenictis*, canids, *Tapocyon*, nimravids).

AC9 (replaces Character 32). Attachment of anterior portion of auditory bulla (cartilaginous or ossified) to squamosal or alisphenoid

0: loose; condition typically indicated by only slight ‘blunting’ of edges of squamosal or alisphenoid on medial or lateral side of Glaserian fissure (e.g. basal carnivoraforms, nimravids and some daphoenines such as *Daphoenus* and *Brachyrhynchocyon*).
1: firm; condition often associated with presence of a deep fossa posterior to postglenoid foramen (e.g. *Paradaphoenus*, *Palaeoprionodon*; cf. Hunt [3,45]).

AC10. Width of basioccipital ventral floor at the level of external auditory meatus

0: less than or equal to width of basicranium lateral to lateral edge of basioccipital ventral floor (e.g. ‘*Miacis*’ *sylvestris*; figure 7*a*)
1: greater than width of basicranium lateral to lateral edge of basioccipital ventral floor (e.g. amphicyonids; figure 7*b*).

## Appendix C. Apomorphies for selected nodes

Characters that are scored for fewer than half of the OTUs in each clade are placed within parentheses. Characters 1–99 are from Wesley-Hunt & Flynn [10] and AC1–AC10 are new to this study; see Material and methods for modifications of character-state definitions.

**Table:**

node	synapomorphy/autapomorphy
A (unambiguous Feliformia)	26 (promontorium apron shelf): 0 (absent) → 1 (small)
	58 (P3 posterior accessory cusp): 2 (absent) → 0 (1 cusp)
	AC5 (p3 posterior cingular cuspulid): 0 (present) → 1 (vestigial/absent)
	AC6 (p4 anterior cuspulid): 0 (small) → 1 (prominent)
	AC8 (m1 hypoconid): 0 (labial) → 1 (lingual)
B	20 (tegmen tympani): 2 (facial nerve partially embedded) → 1 (facial nerve beneath bony sheath)
	53 (M3): 0 (present) → 1 (absent)
	88 (m3): 0 (present) → 1 (absent)
C (Caniformia)	14 (mastoid process direction): 0 (lateral-ventral) → 1 (ventral)
	34 (basioccipital lateral flange): 0 (absent) → 1 (small)
D	AC2 (M1 stylar shelf): 0 (wide) → 1 (narrow)
	AC7 (p4 posterior accessory cuspulid): 0 (absent) → 1 (blade-like)
E	8 (postorbital process): 0 (prominent) → 1 (small)
	14 (mastoid process direction): 1 (ventral) → 3 (swelling)
	25 (internal carotid artery): 0 (transpromontorial & lateral) → 1 (transpromontorial & medial)
	26 (promontorium apron shelf): 0 (absent) → 1 (small)
	33 (shelf between mastoid and paroccipital processes): 0 (wide trough) → 1 (wide but not trough)
	50 (M1 hypocone): 0 (absent) → 2 (swollen lingual cingulum)
F (Amphicyonidae)	(9 (paroccipital process size): 0 (large) → 1 (small))
	24 (suprameatal fossa): 1 (small) → 0 (absent)
	31 (basioccipital excavation): 0 (small) → 2 (extremely deep)
	AC7 (p4 posterior accessory cuspulid): 1 (blade-like) → 2 (step-like)
G	AC1 (M1 lingual cingulum lingual to protocone): 0 (thin band) → 1 (elongate with sharp lingual border)
	AC4 (M2 size): 1 (small-moderate) → 0 (moderate-large)
H	(3 (infraorbital foramen): 1 (round) → 0 (elongate))
	4 (infraorbital foramen): 0 (above P3) → 1 (above P4 anterior edge)
I	82 (P4 protocone): 1 (anterior to paracone) → 0 (medial/posterior to paracone)
J	28 (promontorium anterior shape): 1 (elongate & round) → 3 (elongate & flat)
	40 (middle lacerate foramen): 0 (undefined vacuity) → 1 (posteriorly bordered by promontorium)
K (Canoidea)	4 (infraorbital foramen): 0 (above P3) → 1 (above P4 anterior edge)
	34 (basioccipital lateral flange): 1 (small) → 2 (large)
	53 (M3): 0 (present) → 1 (absent)
	68 (entotympanic): 0 (unossified or loosely attached) → 1 (ossified and firmly attached)
	AC3 (M1 postprotocrista direction): 0 (labial) → 1 (posterior)
	AC9 (anterior bullar attachment): 0 (loose) → 1 (firm)
L (Arctoidea)	AC7 (p4 posterior accessory cuspulid): 1 (blade-like) → 2 (step-like)
	AC10 (basioccipital): 0 (narrow) → 1 (wide)
M (*Cynodictis* spp.)	53 (M3): 0 (present) → 1 (absent)
	55 (P4 parastyle): 0 (absent) → 2 (small bulge)
	AC3 (M1 postprotocrista direction): 0 (labial) → 1 (posterior)
N (*Gustafsonia cognita*)	5 (palatine midline posterior edge): 1 (anterior/equal to tooth row) → 0 (posterior to tooth row)
	15 (condyloid foramen from posterior lacerate foramen): 0 (distant) → 1 (close)
	45 (M1 parastyle direction): 1 (labial) → 0 (anterolabial)
O (*Angelarctocyon australis*)	(none in character matrix)
P (*Paradaphoenus minimus*)	26 (promontorium apron shelf): 1 (small) → 0 (absent)
Q (*Daphoenus* spp.)	(none in character matrix)
R (*Brachyrhynchocyon dodgei*)	(none in character matrix)
S (*Daphoenictis tedfordi*)	25 (internal carotid artery): 2 (not transpromontorial) → 0 (transpromontorial & lateral)
	55 (P4 parastyle): 0 (absent) → 2 (small bulge)
	58 (P3 posterior accessory cusp): 2 (absent) → 1 (2 cusps)
	AC6 (p4 anterior cuspulid): 2 (absent) → 1 (prominent)
T (*Lycophocyon hutchisoni*)	3 (infraorbital foramen): 1 (round) → 0 (elongate)
	13 (mastoid process shape): 1 (swelling) → 0 (extends subequal to or more than paroccipital process)
	49 (M1 paraconule & metaconule): 1 (subequal heights) → 0 (paraconule taller)
	55 (P4 parastyle): 0 (absent) → 2 (small bulge)
